# Airway epithelial ITGB4 deficiency in early life mediates pulmonary spontaneous inflammation and enhanced allergic immune response

**DOI:** 10.1111/jcmm.15000

**Published:** 2020-01-22

**Authors:** Sha Tang, Xizi Du, Lin Yuan, Gelei Xiao, Mengping Wu, Leyuan Wang, ShuangYan Wu, Zhen Duan, Yang Xiang, Xiangping Qu, Huijun Liu, Yizhou Zou, Xiaoqun Qin, Ling Qin, Chi Liu

**Affiliations:** ^1^ Department of Physiology School of Basic Medicine Science Central South University Changsha China; ^2^ Reproductive and Genetic Hospital of Citic‐Xiangya Changsha China; ^3^ Department of Neurosurgery Xiangya Hospital Central South University Changsha China; ^4^ Department of Immunology School of Basic Medicine Science Central South University Changsha China; ^5^ Department of Respiratory Medicine National Clinical Research Center for Respiratory Diseases Xiangya Hospital Central South University Changsha China; ^6^ Research Center of China-Africa Infectious Diseases Xiangya School of Medicine Central South University Changsha Hunan China

**Keywords:** airway epithelial cells, inflammation, integrin β4, thymic stromal lymphopoietin

## Abstract

Lung immune responses to respiratory pathogens and allergens are initiated in early life which will further influence the later onset of asthma. The airway epithelia form the first mechanical physical barrier to allergic stimuli and environmental pollutants, which is also the key regulator in the initiation and development of lung immune response. However, the epithelial regulation mechanisms of early‐life lung immune responses are far from clear. Our previous study found that integrin β4 (ITGB4) is decreased in the airway epithelium of asthma patients with specific variant site. ITGB4 deficiency in adult mice aggravated the lung Th2 immune responses and enhanced airway hyper‐responsiveness (AHR) with a house dust mite (HDM)‐induced asthma model. However, the contribution of ITGB4 to the postnatal lung immune response is still obscure. Here, we further demonstrated that ITGB4 deficiency following birth mediates spontaneous lung inflammation with ILC2 activation and increased infiltration of eosinophils and lymphocytes. Moreover, ITGB4 deficiency regulated thymic stromal lymphopoietin (TSLP) production in airway epithelial cells through EGFR pathways. Neutralization of TSLP inhibited the spontaneous inflammation significantly in ITGB4‐deficient mice. Furthermore, we also found that ITGB4 deficiency led to exaggerated lung allergic inflammation response to HDM stress. In all, these findings indicate that ITGB4 deficiency in early life causes spontaneous lung inflammation and induces exaggerated lung inflammation response to HDM aeroallergen.

## 1 INTRODUCTION

Asthma is a chronic airway inflammation disease characterized by aberrant immune responses to contact aerosol allergen and other environmental insulants.^1^, ^2^ Accumulating evidence shows that the lung immune responses to inhaled allergens are initiated early in embryonic development, in utero, and during infancy.^3^, ^4^, ^5^ The incidence of childhood asthma is lower in infants exposed to large numbers of bacterial and fungal microbes, indicating that early environmental exposures contribute to asthma prevention.^6^ It has been verified that subsequent development of asthma was predicted by the rhinovirus (RV) infection of infants.^7^ The acquisition of T‐cell immunity in infancy strongly influences host defence and allergic reactions, which also mediate subsequent immune responses to external stimuli throughout the life.^8^ However, the nature and regulatory mechanisms of how the early‐life exposures to stimuli instruct the late lung immune response is still unclear.

The airway epithelium is the first cell barrier to allergic stimuli and outside pollutants. It is not only a mechanical physical barrier and a passive target, but also a protector for the lung microenvironment to regulate the immune response.^9^ Accumulating evidence has demonstrated that airway epithelial cells act as a key element in the development of allergy immune response, particularly in asthma exacerbation.^10^, ^11^, ^12^ Stimulation from outside virus, HDM or some other pathogenic particles can directly activate the PRRs (pattern recognition receptors) expressed in the airway epithelium. Then, activated epithelial cells produce and release a variety of cytokines and chemokines, which further cause the activation of inflammatory cells and influence the outcome of the lung immune response.^13^, ^14^

The airway epithelium in asthma patients is often disrupted with impaired physical structure and destroyed functional homeostasis. The onset and clinical phenotype of asthma are associated with disrupted airway epithelial barrier function.^12^, ^15^ Integrins play important roles in the adhesion, tissue repair and microenvironment homeostasis of airway epithelial cells.^16^ Among these molecules, ITGB4 has been shown to decrease significantly in the airway epithelial cells of asthma patients with specific variant sites.^17^, ^18^ By binding with basement membrane component laminin‐5, ITGB4 mediates the structural adhesion of airway epithelial cells to basal surface in hemi‐desmosome structure.^19^ ITGB4 has a unique long cytoplasmic domain subunit that could recruit a range of signalling molecules. Both intracellular phosphatidylinositol 3‐kinase (PI3‐K)^20^ and NF‐κB^21^ signalling pathways could be activated by ITGB4, which indicate that ITGB4 potentially contributes to the development of subsequent immune response. We have also demonstrated that epithelial ITGB4 deficiency aggravated the Th2 inflammation and AHR in an adult house dust mite (HDM)‐induced mouse model.^22^ However, it is still obscure about the contribution of ITGB4 to the lung immunity response following birth. Therefore, this study was to further reveal the role of ITGB4 in the regulation of immune response in early life.

Using our well‐established conditional knockout in vivo system, we demonstrated that airway epithelial postnatal ITGB4 deficiency mediates regulate spontaneous lung inflammation response with ILC2 activation and infiltration of eosinophils and lymphocytes. In neonatal mice, ITGB4 deficiency regulated TSLP production in airway epithelial cells through EGFR pathways. Neutralization of TSLP inhibited the spontaneous inflammation significantly. After HDM stress, ITGB4 deficiency led to exaggerated lung allergic inflammation response. These results indicate that ITGB4 deficiency causes spontaneous lung inflammation during early life and induces exaggerated lung inflammation response to HDM aeroallergen.

## 2 MATERIALS AND METHODS

### 2.1 Animals

The animal studies were approved by the Xiangya Animal Protection and Use Committee of Central South University (No. 201803079). Briefly, the CCSP–rtTA ^tg/−^/TetO‐Cre ^tg/−^/ITGB4 ^fl/fl^ triple transgenic mice were generated by breeding ITGB4^fl/fl^ mice with CCSP–rtTA^tg/−^/TetO‐Cre^tg/tg^ mice.^23^ To delete ITGB4 in early life, 1% Dox (Doxycycline) was placed on the dams of CCSP–rtTA ^tg/−^/TetO‐Cre^tg/−^/ITGB4^fl/fl^ mice from E16.5 to P15.^24^ Under the same Dox induction, the male CCSP–rtTA^tg/−^/TetO‐Cre^tg/−^/ITGB4^fl/fl^ neonatal mice were used as ITGB4^−/−^ mice, the neonatal male littermates lacking either TetO‐Cre, CCSP–rtTA or both were used as ITGB4^+/+^ mice. Asthma was more prevalent among children under 5 years old.^25^, ^26^ Therefore, the age of the transgenic mice in this study ranged from P15 to P18, which probably correspond to the age of children from 3 to 5 years old.^27^, ^28^

### 2.2 HDM challenge and administration of anti‐TSLP neutralizing antibody

For the induction of HDM challenge, mice were intranasally (i.n.) exposed to 50μg HDM on P15, P16 and P17 as previously described.^24^ Non‐sensitized mice were i.n. treated with phosphate‐buffered saline (PBS). The mice were injected ip with 50 µg anti‐TSLP Ab (clone 28F12; eBioscience) to block the effect of TSLP on P3.

### 2.3 Lung histology and immunohistochemical staining

Paraffin‐embedded lung sections were stained with haematoxylin and eosin (HE) (Sigma). Histopathology (inflammatory infiltrates) was scored blindly at morphological criteria.^29^ Expression of ITGB4 on airway epithelial cells was detected by immunofluorescent on lung paraffin sections with the following antibodies: CCSP (sc‐365992, Santa Cruz), ITGB4 (Ab182120, Abcam). Immunohistochemical staining was performed to detect the expression of TSLP on lung paraffin sections with the use of the following antibody: TSLP (ab188766, Abcam). We used Zeiss Discovery.V8 Stereo microscopes (Carl Zeiss MicroImaging GmbH) and Axio‐Cam ICc3 camera (Spectra Service) to photograph. Zeiss AxioVision Rel. 4.7 software was used to get images.

### 2.4 Measurement of airway resistance

Airway resistance after the administration of each dose of methacholine (Sigma‐Aldrich) was assessed with a direct plethysmography (Biosystems XA; Buxco Electronics), as previously described.^30^ In brief, mice were anaesthetized with ketamine (100 mg/kg) and a mixture containing xylazine (10 mg/kg) by ip injection (ratio 1:4, respectively; 150 µL per mouse). A cannula was then inserted into the trachea, and all the mice were ventilated with a tidal volume of 8 mL/kg at a rate of 145 breaths/min. Increasing concentrations of aerosolized methacholine (1.56‐25 mg/mL) were delivered intratracheally. AHR was presented as increase of percentage above the baseline (saline challenge).

### 2.5 Bronchoalveolar lavage fluid (BALF) collection and cell counting

Bronchoalveolar lavage fluid was collected and processed as described previously.^31^ In brief, the lung was lavaged with 0.5 mL ice‐cold PBS containing 0.1 mmol/L EDTA twice. Red blood cells were removed by use of hypotonic red blood cell lysis buffer. Then, BALF was centrifuged to collect cellular infiltrate. The total number of cells was quantified with a haemocytometer, and the cells were plated on a glass slide. The differential leucocyte counts were determined at morphological criteria by light microscopy (×100) on May‐Grunwald and Giemsa‐stained slides.

### 2.6 Flow cytometry

Single‐cell suspensions from lungs were prepared for flow cytometric analysis as previously described.^32^, ^33^ Briefly, lung tissue was enzymatically digested with 5,000 caseinolytic units of dispase (Discovery Labware Inc, Corning) to prepare cell suspensions. After that, collected cells were stimulated with PMA (50 ng/mL) and ionomycin (500 ng/mL) in the presence of brefeldin A (5 mg/mL) for 4 hours at 37°C. Then, fluorochrome‐labelled antibodies were used to stain single‐cell suspensions.

The fluorescence staining programme was performed as previously described.^34^, ^35^ Briefly, cells were incubated with fixable viability dye (BD Biosciences) for 15 minutes in the dark on ice to exclude the dead cells at first. Then, cells were stained with FITC‐conjugated anti‐CD45, BV421‐conjugated anti‐CD4, BV510‐conjugated anti‐CD3 and isotype controls (BD Biosciences) to obtain Th cells subgroup. Subsequently, cells were fixed with 4% PFA and permeabilized with Cytofix/Cytoperm solution (BD Biosciences). After that, different T‐cell groups were then stained with APC‐conjugated anti‐IFN‐γ, PE‐conjugated anti‐IL‐4 and PE‐cy7‐conjugated anti‐IL‐17A antibodies (BD Bioscience) to analyse the derivation of Th cells. For analysis of Tregs, APC‐conjugated anti‐CD25 was added in the surface marker staining step. Then, cells were fixed and permeated with Foxp3/Transcription Factor Fix & Perm Kit (Lianke bio) and PE‐conjugated antimouse Foxp3 (BD Biosciences). For analysis of ILC2, cells were sorted as Lin^−^CD45^+^Thy1.2^+^CD25^+^ (BD Biosciences).

### 2.7 ELISA assay

ELISA Kit (RAB0504‐1, Sigma) was used to determine the levels of TSLP according to the manufacturer's protocols.

### 2.8 Primary airway epithelial cells

Airway epithelial cells were isolated from mouse lung as previously described.^36^ CCSP^+^ bronchial epithelial cells were sorted by flow cytometry with CCSP (sc‐365992, Santa Cruz). CCSP^+^ bronchial epithelial cells from ITGB4^−/−^ mice were cultured and used as ITGB4 deleted bronchial epithelial cells.

### 2.9 Quantitative RT‐PCR

Total RNA was prepared with TRIzol reagent (Invitrogen) and phenol‐chloroform extracts from primary airway epithelial cells and quantified on a SmartSpec™ Plus spectrophotometer (Bio‐Rad).^37^ RT‐PCR was conducted to synthesize cDNA by use of oligo d(T) primer (Invitrogen) on a T100 thermal cycler (Bio‐Rad). Quantitative PCR (qPCR) was performed on a CFX96 Touch™ Deep Well Real‐Time PCR Detection System (Bio‐Rad) using TaqMan Gene Expression Master Mix (Applied Biosystems) with thermal cycling conditions. Primer sequences were described in Table 1.

**Table 1:** TaqMan primers for qRT‐PCR

Mouse gene in Taqman expression assay	Primer sequence
IL‐25	Forward: 5′‐CAGCAAAGAGCAAGAACC‐3′
	Reverse: 5′‐CCCTGTCCAACTCATAGC‐3′
IL‐33	Forward: 5′‐ATGCTGGATTGCAGAGCAGTA‐3′
	Reverse: 5′‐ACGGGGCACATTATTTTTAGTCT‐3′
TSLP	Forward: 5′‐ACGGATGGGGCTAACTTACAA‐3′
	Reverse: 5′‐AGTCCTCGATTTGCTCGAACT‐3′
β‐actin	Forward: 5′‐TTGCAGCTCCTTCGTTGCC‐3′
	Reverse: 5′‐GACCCATTCCCACCATCACA‐3′

### 2.10 Immunoprecipitation

In brief, protein was extracted with radioimmunoprecipitation assay (RIPA) buffer: 50 mmol/L Tris‐HCl, 1% NP40, 0.5% sodium deoxycholate, 150 mmol/L NaCl, 0.1% SDS, 5 mmol/L EDTA, pH = 7.4.^38^ The immune complexes were allowed to bind to Protein A‐Sepharose beads for 3 hours at 4°C, washed (15 minutes, 4°C) with RIPA buffer and resuspended in loading buffer. Immunoprecipitates were then subjected to 10% SDS‐PAGE and immunoblotted on a polyvinylidene difluoride membrane. Immunocomplexes were stained with anti‐ITGB4 (Ab197772, Abcam) as well as EGFR (Ab52894, Abcam) by use of ECL (NCI4106, Thermo Pierce).

### 2.11 Western blot

Primary airway epithelial cells were harvested and extracted for protein as previously described.^39^ Then, 50 µg protein was separated by 10% SDS‐PAGE and transferred to a polyvinylidene difluoride membrane. Some epithelial cells were pretreated with AG1478 (1 µmol/L, an EGFR phosphorylation inhibitor). Levels of ITGB4, TSLP, EGFR and phosphorylated EGFR (p‐EGFR) were determined respectively with antimouse antibodies against these proteins as previously reported.

### 2.12 Data analysis

All data were analysed with GraphPad Prism Software (Version 6; San Diego, CA) and presented as mean ± SEM. Two‐way ANOVA followed by Dunnett's post hoc test was used to identify differences in lung function. Student's unpaired *t* test was used for all other comparisons. Differences were considered statistically significant for *P < .05, **P < .01 or ***P < .001.

## 3 RESULTS

### 3.1 Construction of neonatal mice with ITGB4‐specific deficiency

**Figure 1:**
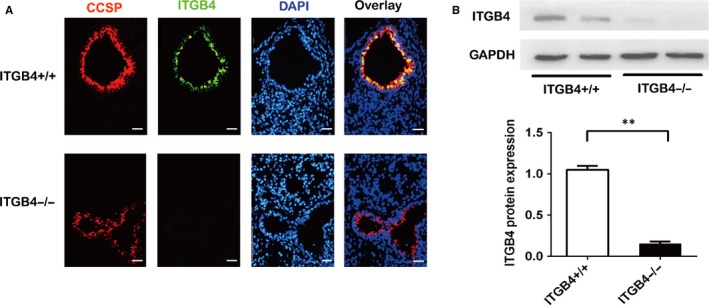
Expression of ITGB4 in airway epithelial cells was deleted from ITGB4^–/–^ mice on P15. A, ITGB4 deficiency validation was detected on P15 via immunofluorescence. Colocalization of ITGB4 and CCSP was observed in lung sections. ITGB4 and CCSP were stained with green and red fluorescent separately. Cell nuclei were stained with DAPI (blue), bars: 25 µm. B, ITGB4 protein expression from CCSP^+^ airway epithelial cell was detected by Western blot (n = 6). Values represented as mean ± SEM. **P < .01 by an unpaired, Student's *t* test

ITGB4 protein was conditionally deleted in CCSP^+^ airway epithelial cells as induced by Dox administration. Dual immunofluorescence staining was used to detect the specific localization and expression of ITGB4 in lung tissue. In control mice, ITGB4 was expressed in near‐linear basilar stained airway epithelial cells along the conducting airways. However, in the ITGB4^−/−^ mice, ITGB4 was deleted significantly in the conducting bronchi and proximal bronchioles (Figure 1A). Besides, the expression of ITGB4 in colonic epithelial cells and skin keratinocytes was also detected on P15 (Figure S1) which further verifies the specific deletion of ITGB4 in airway epithelial cells. In addition, primary CCSP^+^ airway epithelial cells were also used to detect ITGB4 expression by Western blot staining which showed an obviously apparent ITGB4 deletion in ITGB4^−/−^ mice (Figure 1B).

### 3.2 ITGB4 deficiency caused pulmonary spontaneous inflammation and AHR in neonatal mice

**Figure 2:**
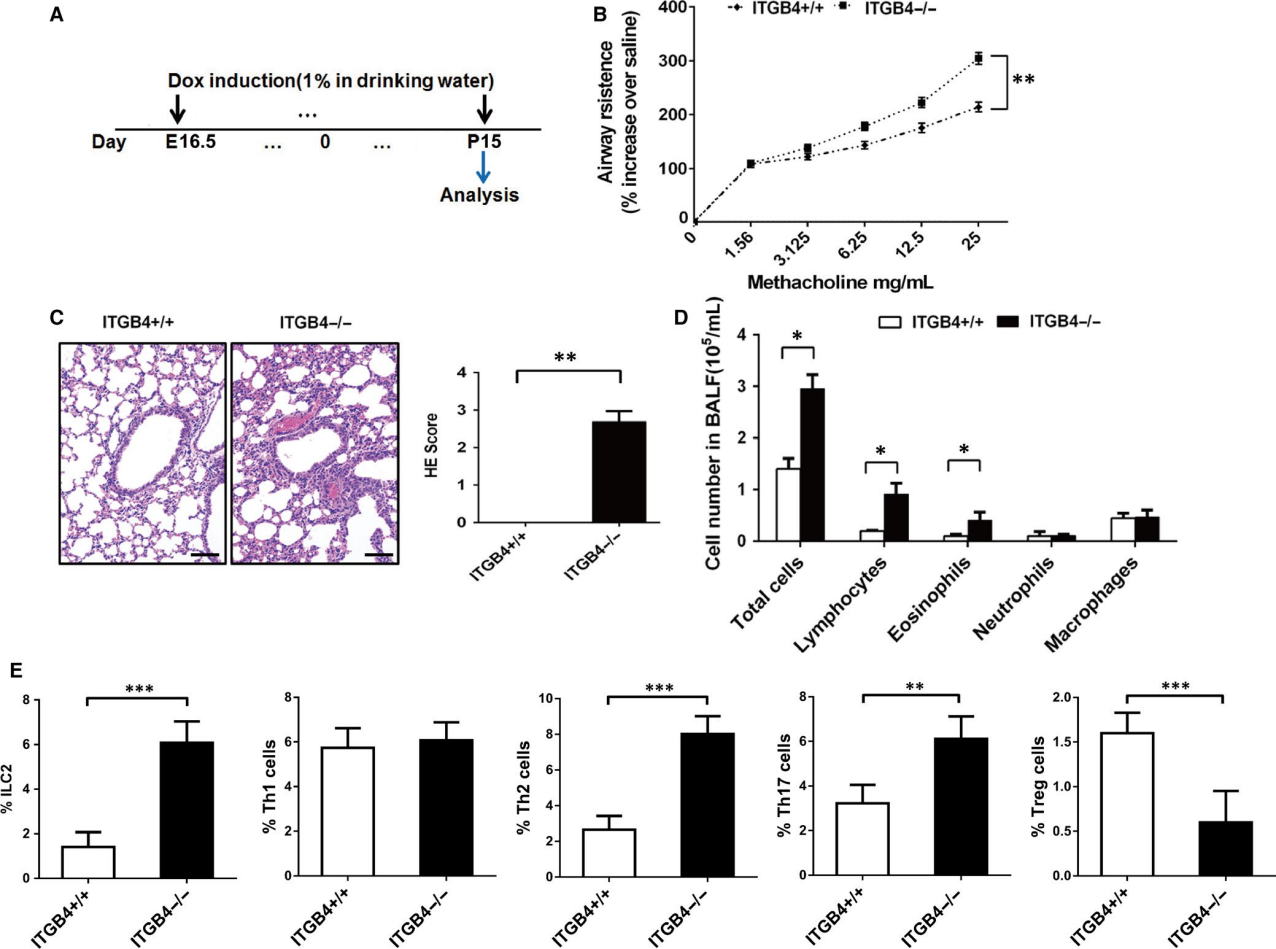
ITGB4 deficiency caused lung inflammation and AHR in neonatal mice. A, Dox was placed on the dams in drinking water from E16.5 to P15. B, Analysis of AHR and lung inflammation was conducted at P15. Lung resistance was determined by administering ascending doses of methacholine. The response to each dose of methacholine was quantified for airway mechanics parameter as the average of the 4 peak measurements. Data represent the mean ± SEM of six mice per group. **P < .01 by 2‐way ANOVA followed by Fisher post hoc test. C, Lung histopathological assessment was observed by HE staining (n = 8), bars: 50 µm. Values represented as mean ± SEM. **P < .01 compared with controls using an unpaired, Student's *t* test. D, BALF inflammatory cell counts were determined (n = 8). Values represented as mean ± SEM. *P < .05 compared with controls using an unpaired, Student's *t* test. E, The infiltration of ILC2, Th1, Th2, Th17 and Treg cells in the lung of ITGB4^+/+^ and ITGB4^–/–^ mice was detected by flow analysis (n = 10). Values represented as mean ± SEM. **P < .01 or ***P < .001 compared with controls using an unpaired, Student's *t* test

Dox‐induced timeline was shown in a pictorial timeline (Figure 2A). The influence of ITGB4 deletion in the regulation of pulmonary inflammation and AHR was assessed on P15. Compared with ITGB4^+/+^ mice, AHR to methacholine was obviously increased in ITGB4^−/−^ mice (Figure 2B). Meanwhile, ITGB4 deficiency increased inflammatory infiltrates, which is different from the lack of inflammation in the lung of ITGB4^+/+^ mice (Figure 2C). Consistent with the observed inflammation in lung tissue, increased lymphocytes and eosinophils infiltrated into the BALF of ITGB4^−/−^ mice. And the infiltrated inflammation cells were primary lymphocytes which had a 3.7‐fold growth (Figure 2D). In order to more precisely interpret the impact of ITGB4 deletion on the activation of lymphocyte differentiation subgroups, we further examined the infiltration of ILC2, Th1, Th2, Th17 and Treg cells by flow cytometry. Increased ILC2, Th2, Th17 and decreased Treg cells were induced in the lung of ITGB4^−/−^ groups compared with ITGB4^+/+^ control groups (Figure 2E and Figure S2).

### 3.3 Increased expression of TSLP in ITGB4‐deficient airway epithelial cells

**Figure 3:**
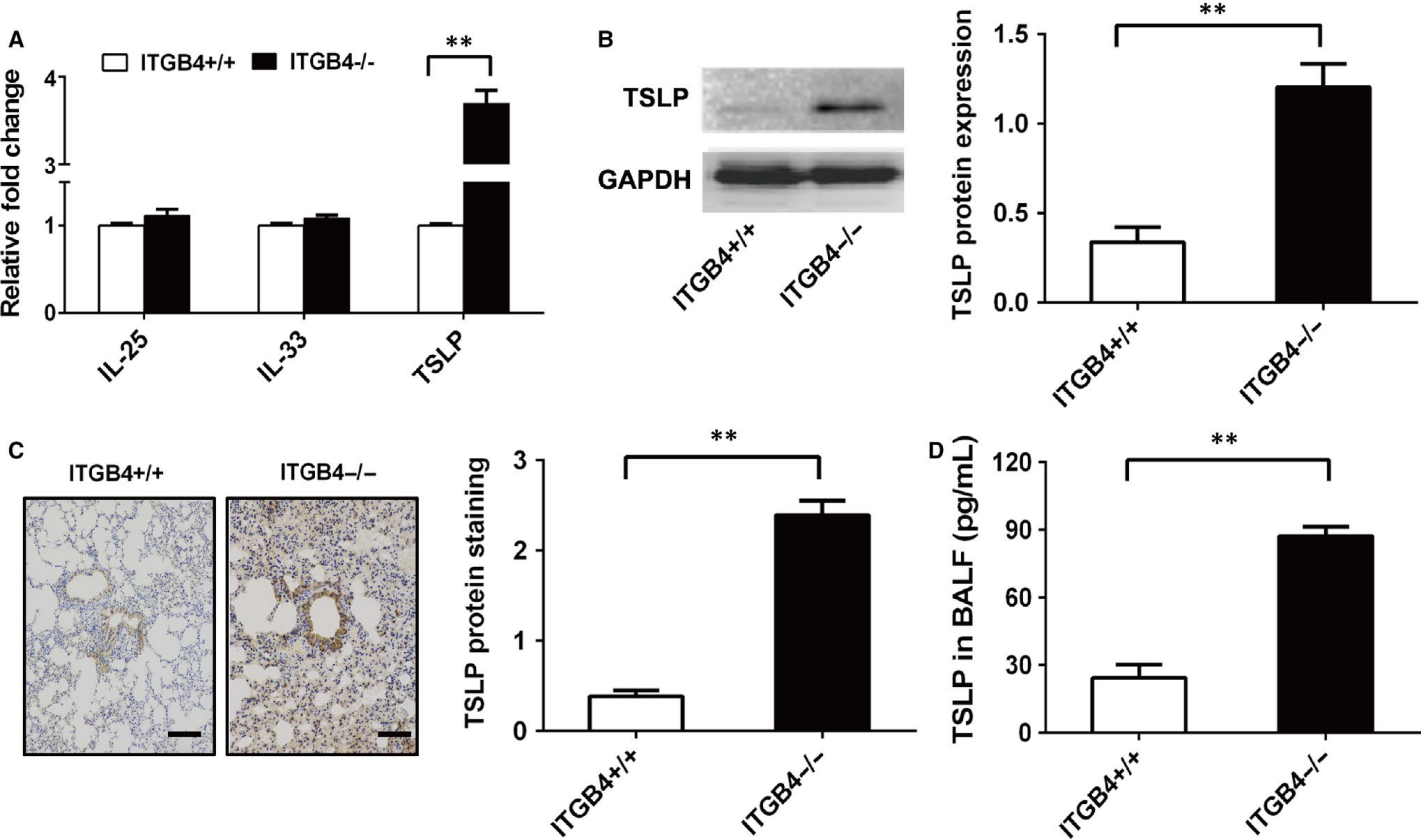
ITGB4 deficiency leads to increased secretion of TSLP from primary airway epithelial cells on P15. A, Primary airway epithelial cells were isolated from the lung of ITGB4^+/+^ or ITGB4^−/−^ mice on P15. The level of IL‐25, IL‐33 and TSLP transcription in airway epithelial cells (n = 10) was detected by qPCR. Values represented as mean ± SEM. **P < .01 compared with controls using an unpaired, Student's *t* test. B, Western blot staining of airway epithelial cells for TSLP. Values represented as mean ± SEM for six samples from one experiment and representative of 3 independent experiments. **P < .01 using an unpaired, Student's *t* test. C, TSLP expression in airway mucus was detected by immunohistochemistry. D. TSLP protein in BALF (n = 8) was determined by ELISA. Values represented as mean ± SEM. **P < .01 compared with controls using an unpaired, Student's *t* test

ILC2 initiates and maintains the adaptive Th2 immune response which can be activated by IL‐25, IL‐33 and TSLP.^40^ To determine how ITGB4 contributes to the activation of ILC2, we examined the expression of IL‐25, IL‐33 and TSLP in ITGB4‐deficient airway epithelial cells. Significantly, higher levels of TSLP transcription were detected in the primary airway epithelial cells of ITGB4^−/−^ mice, as compared to ITGB4^+/+^ mice. While, no significant difference was detected in the transcription levels of IL‐25 and IL‐33 (Figure 3A). Consistent with increased TSLP mRNA expression, TSLP protein expression in ITGB4‐deficient airway epithelial cells also increased significantly (Figure 3B). Meanwhile, higher level of TSLP expression in lung tissue (Figure 3C) and secretion in BALF (Figure 3D) was also detected in ITGB4^−/−^ mice, as compared with ITGB4^+/+^ mice.

### 3.4 ITGB4 regulates TSLP expression through the activation of EGFR pathway

**Figure 4:**
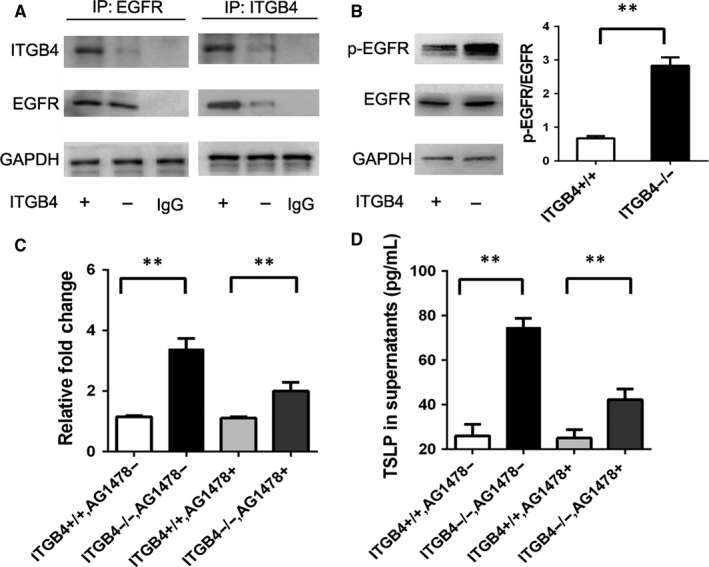
ITGB4 deficiency induced increased higher EGFR phosphorylation which further induced the expression of TSLP. A, Interaction between ITGB4 and EGFR was assessed by immunoprecipitation on airway epithelial cells. B, EGFR and phosphor EGFR were detected by Western blot from ITGB4^–/–^ cells and control cells. Values represented as mean ± SEM for six samples from one experiment and representative of 3 independent experiments. **P < .01 using an unpaired, Student's *t* test. C, TSLP mRNA expression was detected from ITGB4^–/–^ cells and control cells (n = 8) in the presence of EGFR phosphorylation inhibitor AG1478. Values represented as mean ± SEM. **P < .01 compared with controls using an unpaired, Student's *t* test. D, The secretion of TSLP in culture supernatant (n = 8) was detected from ITGB4^–/–^ cells and control cells by ELISA after AG1478 treatment. Values represented as mean ± SEM. **P < .01 compared with controls using an unpaired, Student's *t* test

Integrin β4 has shown to interact with EGFR in a ligand‐independent manner and mediate the activation of EGFR pathway.^41^, ^42^, ^43^ The expression of TSLP was regulated through EGFR transactivation in human and mouse keratinocytes.^44^ Then, we want to determine the possibility of the involvement of EGFR‐mediated signalling pathways in ITGB4‐regulated TSLP production. The interaction between ITGB4 and EGFR in airway epithelial cells was detected by immunoprecipitation (Figure 4A). Meanwhile, Western blot analysis revealed that ITGB4 deficiency induced higher EGFR phosphorylation (Figure 4B). Importantly, the EGFR inhibitor treatment could block the heightened transcription level and secretion of TSLP in ITGB4^−/−^ airway epithelial cells (Figure 4C‐D).

### 3.5 TSLP blocking inhibited the pulmonary inflammation and AHR in ITGB4^−/−^ mice

**Figure 5:**
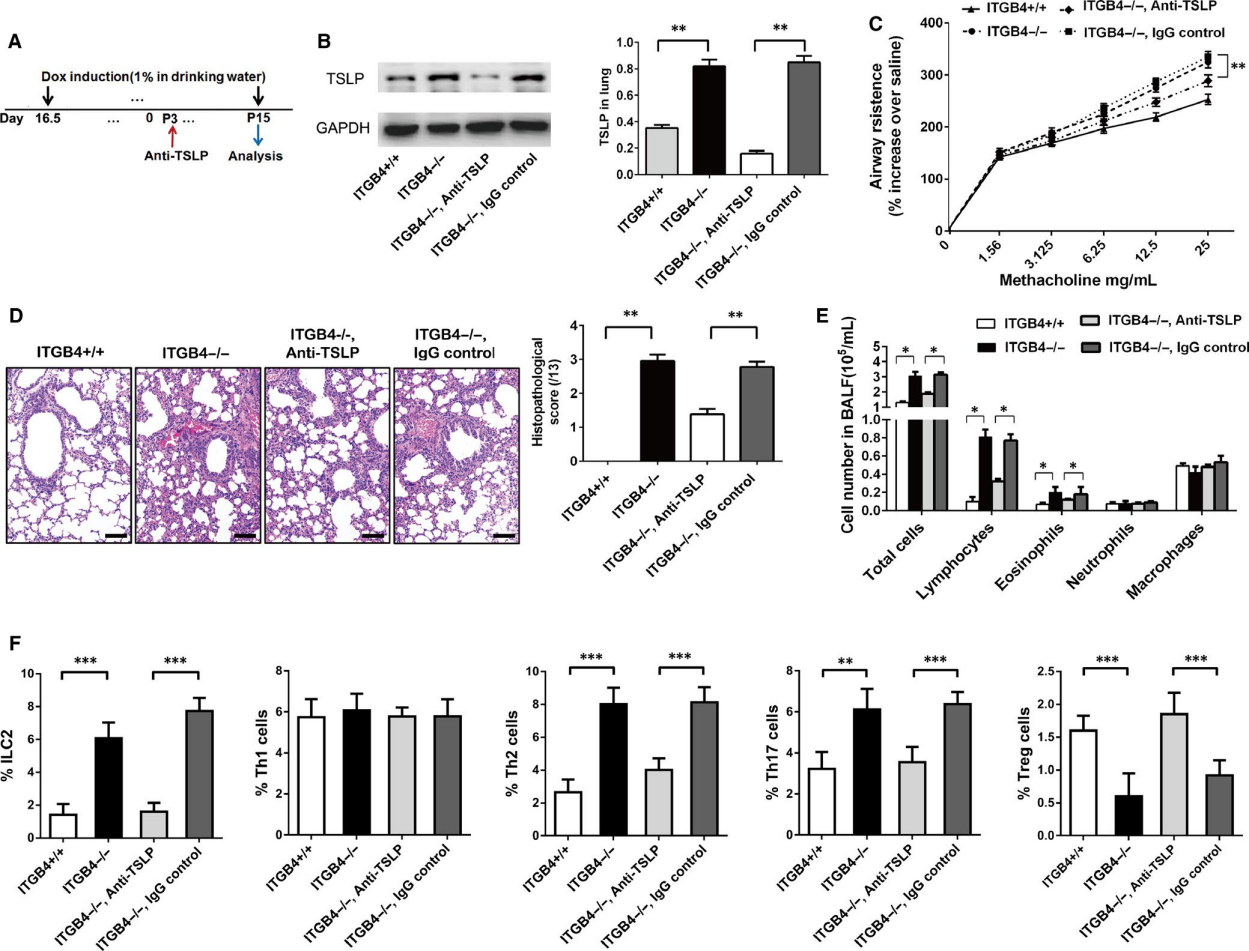
Blockade of TSLP inhibits both ILC2 activation and enhanced AHR, exaggerated lung inflammation in ITGB4^−/−^ mice. A, ITGB4^−/−^ mice received treatment with either anti‐TSLP or isotype control antibodies. B, TSLP protein in lung was determined by Western blot. Values represented as mean ± SEM for 6 samples from one experiment and representative of 4 independent experiments. **P < .01 using an unpaired, Student's *t* test. C, AHR was conducted as airway resistance in response to methacholine. Data represent the mean ± SEM of 7 mice per group. **P < .01 by 2‐way ANOVA followed by Fisher post hoc test. D, Lung histology was assessed with HE staining (n = 8), bars: 50 µm. Values represented as mean ± SEM. **P < .01 compared with controls using an unpaired, Student's *t* test. E, BALF inflammatory cell was counted (n = 8). Values represented as mean ± SEM. *P < .05 compared with controls using an unpaired, Student's *t* test. F, The level of ILC2, Th1 cells, Th2 cells Th17 cells and Treg cells was detected by flow analysis (n = 10). Values represented as mean ± SEM. **P < .01 or ***P < .001 compared with controls using an unpaired, Student's *t* test

Thymic stromal lymphopoietin has been demonstrated to be an important cytokine to promote the activation of ILC2, which plays a key role in the immunopathology of lung immune responses.^45^ Then, TSLP was neutralized to determine whether TSLP neutralization could alleviate the lung inflammation in ITGB4^−/−^ mice. TSLP‐specific antibody was used to block the effect of TSLP (Figure 5A). Increased TSLP levels in lung of ITGB4^−/−^ mice were dramatically contained by anti‐TSLP mAb pretreatment (Figure 5B). Anti‐TSLP mAb alleviated AHR to methacholine obviously (Figure 5C). Meanwhile, TSLP neutralization blocked the spontaneous airway inflammation (Figure 5D) and the infiltration of lymphocytes and eosinophils (Figure 5E). Consistent with the above results, after TSLP blocking, the infiltration of ILC2, Th2 cells and Th17 cells was also inhibited significantly. Meanwhile, Treg cells increased significantly in the ITGB4^−/−^ mice after TSLP neutralization (Figure 5F and Figure S3).

### 3.6 ITGB4 deficiency enhanced airway inflammation and AHR in neonatal mice after HDM challenge

**Figure 6:**
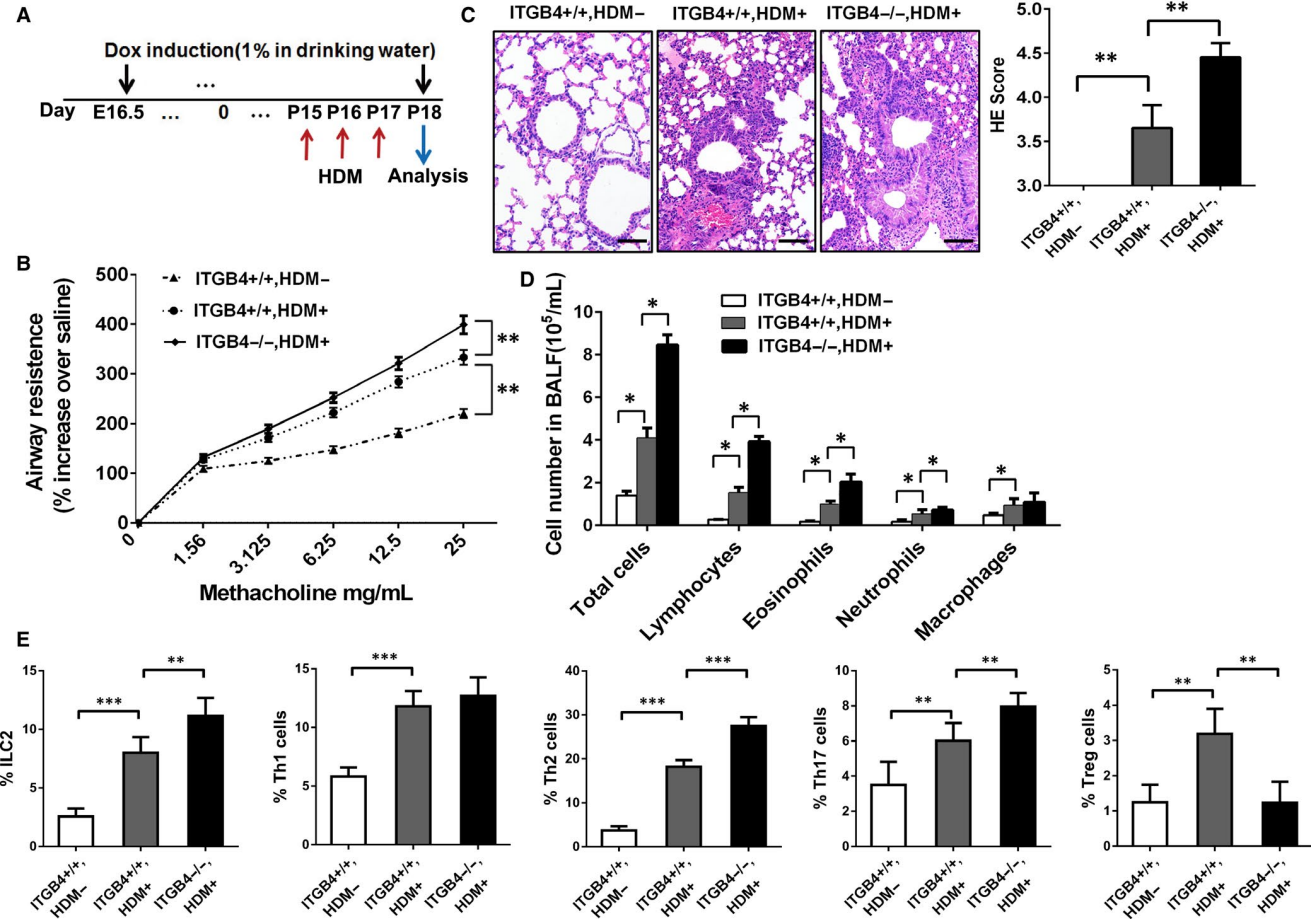
ITGB4 deficiency induced increased lung inflammation and AHR in response to HDM. A, Dox was given in drinking water from E16.5 to P18. HDM was stressed from P15 to P17. Analysis of AHR and lung inflammation was conducted on P18. B, Analysis of AHR was detected by assessing lung resistance after administering ascending doses of methacholine. Data represented the mean ± SEM of 6 mice per group. **P < .01 by 2‐way ANOVA followed by Fisher post hoc test. C, Lung histology was assessed with HE staining (n = 8), bars: 50 µm. Values represented as mean ± SEM. **P < .01 compared with controls using an unpaired, Student's *t* test. D, Inflammatory cell number in BALF was determined. Values represented as mean ± SEM. *P < .05 compared with controls using an unpaired, Student's *t* test. E, After HDM stress, the infiltration of ILC2, Th1 cells, Th2 cells Th17 cells and Treg cells in lung was assessed by flow analysis (n = 10). Values represented as mean ± SEM. **P < .01 or ***P < .001 compared with controls using an unpaired, Student's *t* test

Since ITGB4 deficiency caused spontaneously pulmonary inflammation and AHR after birth, we further detected whether ITGB4 deficiency influenced pulmonary inflammation and AHR after exposure to HDM in the postnatal period. ITGB4^−/−^ mice and control mice were treated with 100 µg HDM extract (i.n.) for 3 days (Figure 6A). After HDM stress, ITGB4^−/−^ mice showed significantly higher AHR than ITGB4^+/+^ mice (Figure 6B). HE staining in lung section showed a severe inflammatory cells infiltration in the lungs of ITGB4^−/−^ mice compared with moderate cellularity in the lungs of ITGB4^+/+^ mice after HDM stress, which was confirmed by the inflammation score (Figure 6C). Consistent with the HE staining results outcome, after HDM challenge, ITGB4 deficiency caused marked extravasation of lymphocytes and mild increase of eosinophils and neutrophils (Figure 6D). The number of ILC2, Th2 and Th17 cells was also increased, and the number of Treg cells was decreased in the lung of ITGB4^−/−^ groups compared with ITGB4^+/+^ groups after HDM stress. While, there is no obvious change in the number of Th1 cells (Figure 6E and Figure S4).

## 4 DISCUSSION

Lung immune response is acquired rapidly after birth and exposure to outside pathogenic microbes, toxicants and allergen that further recruit inflammatory cells to the lung.^6^, ^46^ The airway epithelium is considered to be central to the coordination of pulmonary innate and adaptive immune responses to these challenges.^12^ The present findings demonstrate that airway epithelial ITGB4 deficiency after birth was sufficient to induce spontaneous inflammation and exaggerated allergic immune response in lung, indicating the definite role of ITGB4 in the orchestration of lung immune response in early life.

By the secretion of diverse cytokines and chemokines, airway epithelial cells play a key role in the recruitment and activation of professional immune cells.^12^ Although it is suggested that ITGB4 potentially contributes to the instigation of subsequent immune response, the inner mechanisms are complicated. Interestingly, TSLP, which can be produced by epithelial cells, increased significantly by deficiency of ITGB4 in neonatal mice. Increased TSLP secretion could attract and activate ILC2 which further recruit Th2 cells. Consistent with the specific role of TSLP, anti‐TSLP Ab substantially inhibited the spontaneous lung inflammation in ITGB4‐deficient mice.

ILC2 is distributed widely in the mucosal barriers of the lung, which initiate and maintain the adaptive type 2 immune response in the pathogenesis of allergic asthma.^47^ Unlike adaptive T cells, ILC2 does not express rearranged antigen receptors which can be activated by several specific cytokines.^48^ Roles of these cytokines in ILC2‐dependent inflammation after outside disparate insults were revealed in vitro and in vivo. However, the cellular sources of these signals remain unclear. At the mucosa of the lungs, airway epithelial cells seem to be especially important regulators of ILC2. The complex interplay between ILC2 and epithelial cells has been an area of intense research efforts.^49^, ^50^ Airways epithelium responds to external environmental stimuli by releasing IL‐25, IL‐33 and TSLP which play a pivotal role in ILC2 activation and the initiation of allergic inflammation. Particularly, in the induction of Th2 inflammation in various allergic diseases, TSLP plays a critical role which is mainly produced by epithelial cells.^51^

Increased TSLP was detected in airway epithelia of asthmatic patients, and elevated TSLP is associated with both the expression of Th2‐attracting chemokines and the disease severity.^52^ Besides, TSLP levels in BALF are associated with corticosteroid resistance in ILC2 in asthmatic patients.^53^ However, the underlying regulation mechanisms of TSLP on airway epithelial cells remain unclear. Since the fundamental findings of Moro et al^54^ who first showed transactivation of EGFR by integrins, a huge body number of researches work has have confirmed this pathway.^55^, ^56^ ITGB4 is also observed to interact with EGFR in a ligand manner in hepatocellular carcinoma cell line.^41^ ITGB4 and EGFR have previously been demonstrated to colocalize, which impairs the mobility of EGFR in the plasma membrane and reduces the binding efficiency of the ligand to EGFR.^57^ Our findings are consistent with these previous publications, that is, ITGB4 interacts directly with EGFR in airway epithelial cells. Our results are in line with these previous findings, we observed the direct interaction between ITGB4 and EGFR in airway epithelial cells. TSLP expression was verified to be regulated through EGFR transactivation in human and mouse keratinocytes.^44^ We also found that ITGB4 deficiency increased TSLP expression due to enhanced phosphorylation of EGFR pathway. Thus, these results relate ILC2 activation with epithelial cells that monitor epithelial integrity and regulate lung inflammation. Furthermore, we also found that ITGB4 deficiency caused aggravated lung inflammatory responses after HDM challenge in postnatal mice. It means that, besides a traditional structural adhesion molecule, ITGB4 can also act as anti‐inflammatory protein on airway epithelial cells. HDM activates airway inflammation through TLR4, which is required for normal Th2 responses in the lungs.^58^, ^59^ Based on ITAM (immunoreceptor tyrosine‐based activation motif)‐associated receptors, a series of elegant experiments demonstrate the roles of integrins in the negative regulation of TLR4 signalling pathway.^60^, ^61^ It has also defined that ITGB4 regulates early signalling molecule Src which could inhibit the activation of TLR4‐triggered innate inflammatory responses yy.^62^, ^63^ Whether the mechanism of the anti‐inflammatory effect of ITGB4 takes place through regulation of TLR4 requires further verification and studies on other alternative pathways.

Although our results provide that epithelial ITGB4 mediates pulmonary spontaneous inflammation and aggravated allergic immune response in early life, there are some limitations. The first one is that further lung insult exploration is needed to detect in ITGB4 deficiency mice, because it may also cause spontaneous inflammation. Besides, the regulation mechanism of ITGB4 deficiency in airway epithelial cells is still obscure. In addition, the effect of ITGB4 recovery on airway immune response should also be demonstrated in subsequent studies.

In summary, we demonstrated that ITGB4 deficiency induces spontaneous lung inflammation and enhanced allergic lung immune response after birth. The specific immune responses after birth may have long‐term effects on the pattern of subsequent immune and inflammatory responses in the lung. These progresses in this study further help us understand how epithelial cell dysfunction contributes to the pathogenesis of asthma immune responses in early life.

## CONFLICT OF INTEREST

The authors declare that they have no conflict of interest.

## AUTHOR CONTRIBUTIONS

CL and LQ conceived and designed this study. ST, XZD LY, LYW, SYW and MPW carried out the experiments. YX, XPQ, HJL, GLX, ZZ and YZZ contributed to the interpretation of the results findings. CL and XQQ wrote the paper. All authors provided critical feedbacks and helped shape the research, analysis and manuscript.

## Data Availability

All data used and analysed during this study are included in this article are available.
