# Supplementary figures and images for: Airway epithelial ITGB4 deficiency in early life mediates pulmonary spontaneous inflammation and enhanced allergic immune response
Source: J Cell Mol Med. 2020 Jan 22;24(5):2761–71. doi: 10.1111/jcmm.15000 (PMC7077534; doi:10.1111/jcmm.15000)

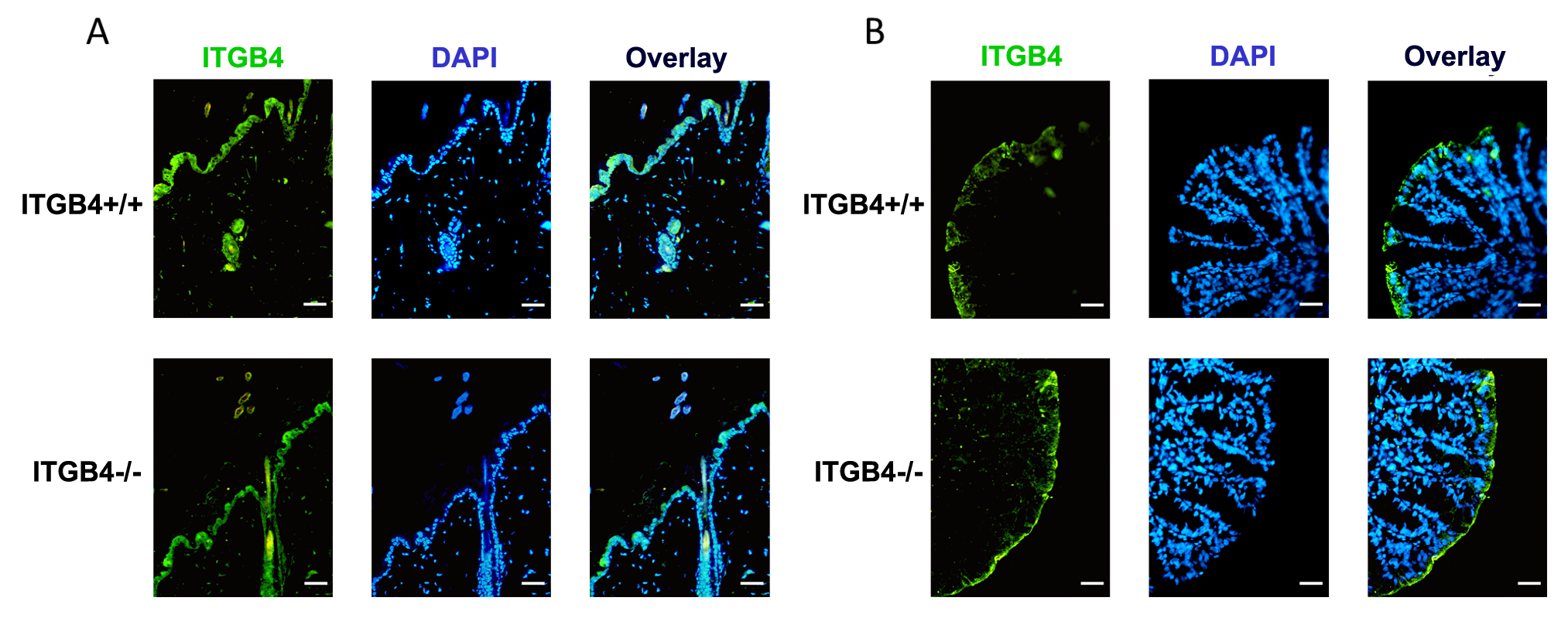

Supplement: Supplementary file 1 [file JCMM-24-2761-s001.tif]

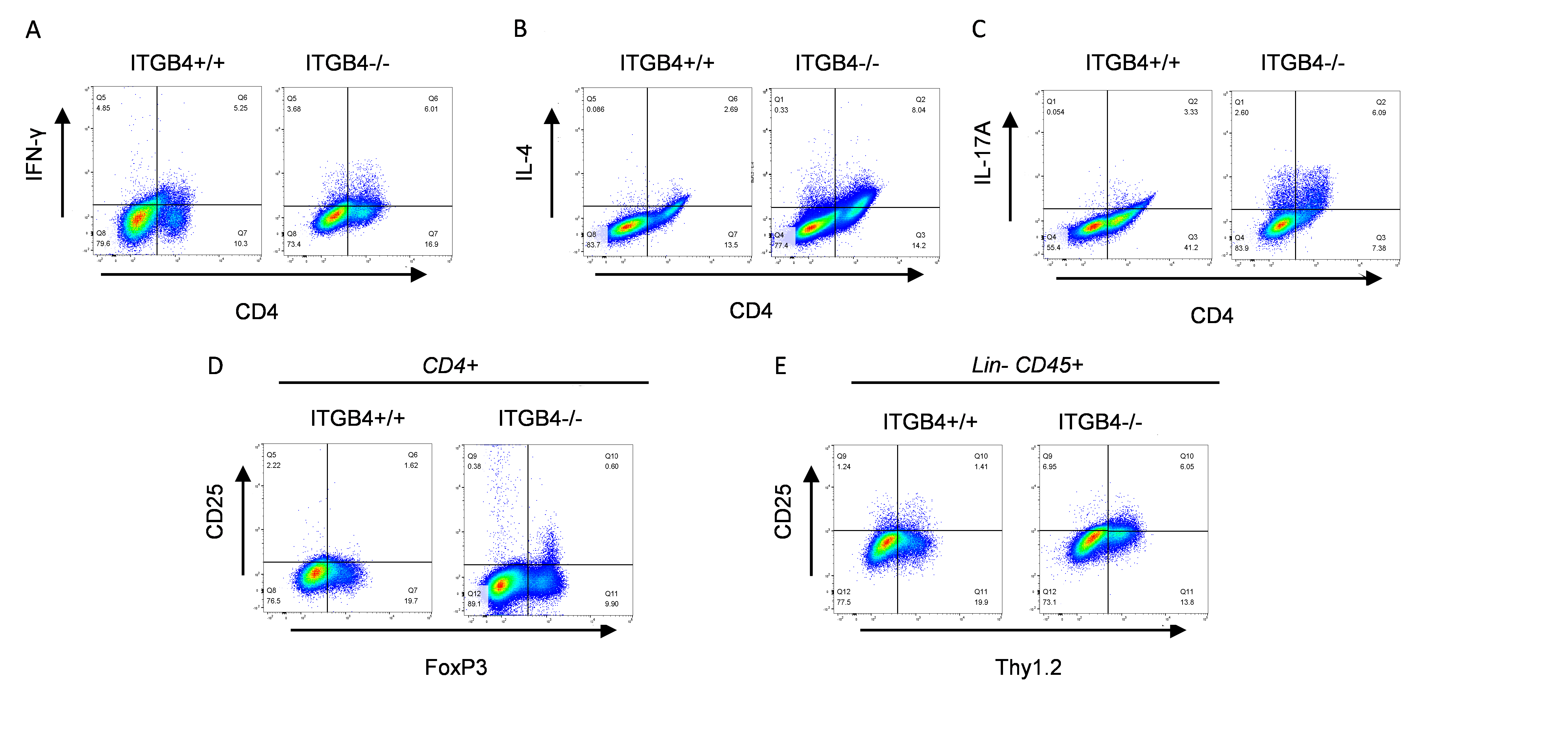

Supplement: Supplementary file 2 [file JCMM-24-2761-s002.tif]

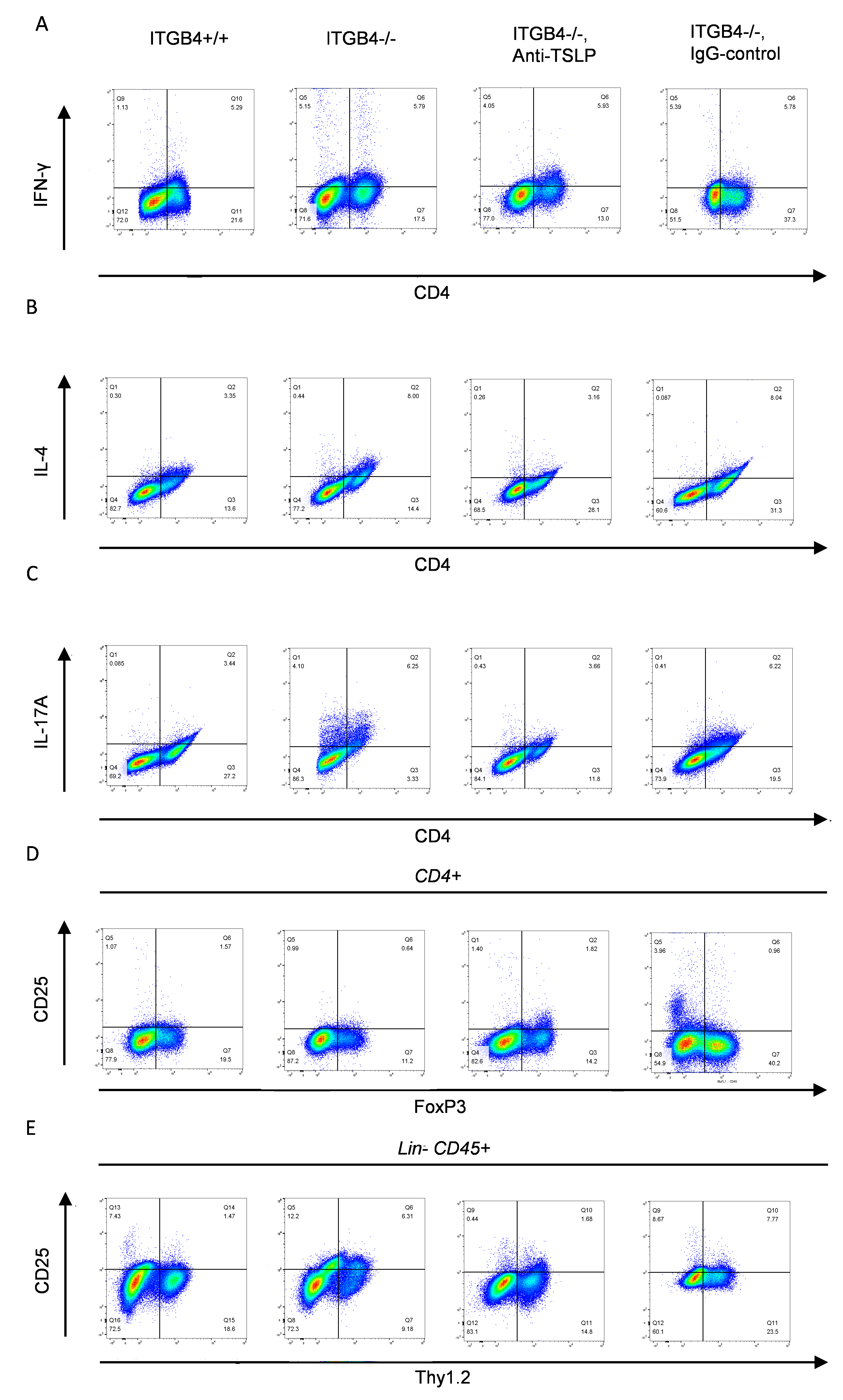

Supplement: Supplementary file 3 [file JCMM-24-2761-s003.tif]

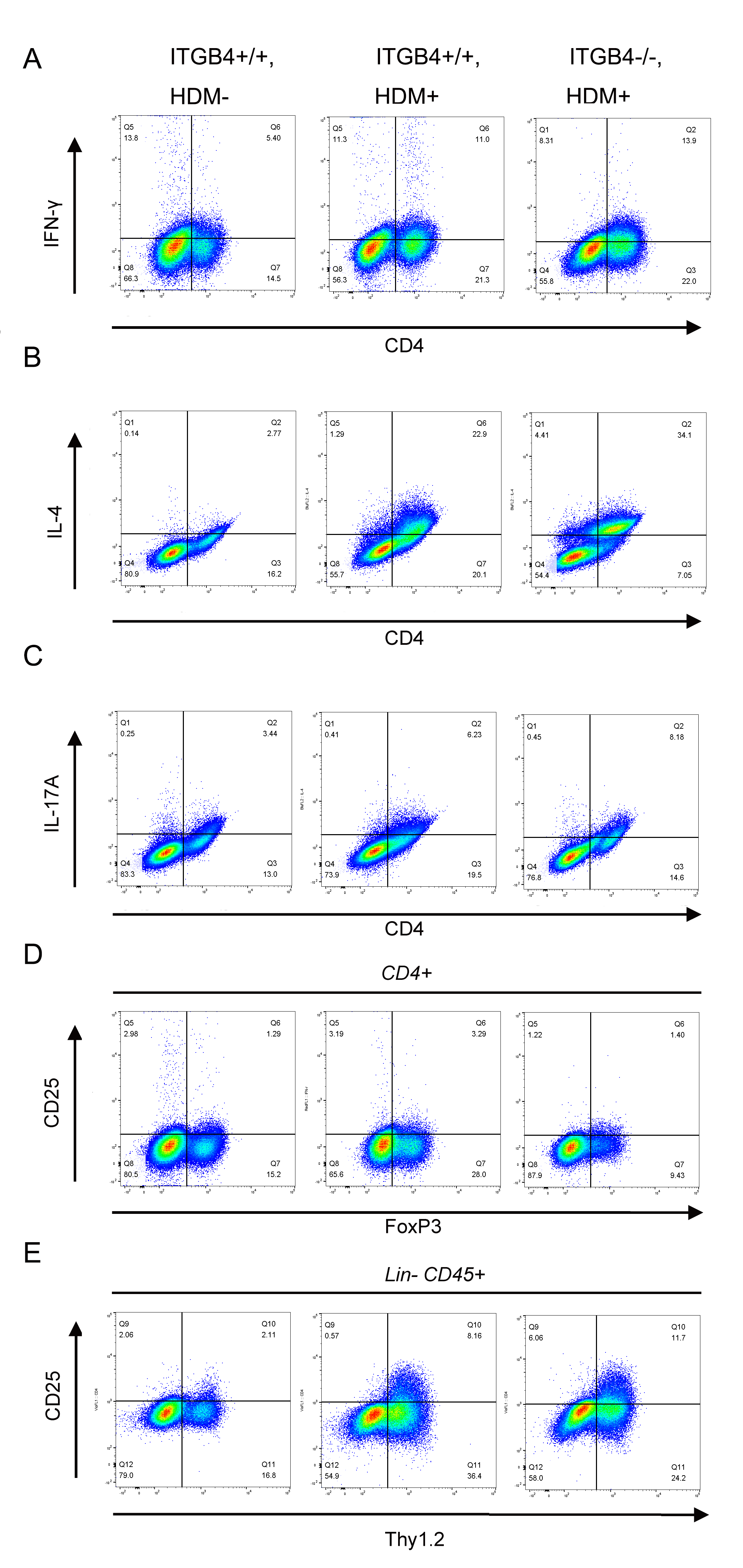

Supplement: Supplementary file 4 [file JCMM-24-2761-s004.tif]
